# Temporal effect of HLA-B*57 on viral control during primary HIV-1 infection

**DOI:** 10.1186/1742-4690-10-139

**Published:** 2013-11-18

**Authors:** Sagar A Vaidya, Hendrik Streeck, Noor Beckwith, Musie Ghebremichael, Florencia Pereyra, Douglas S Kwon, Marylyn M Addo, Jenna Rychert, Jean-Pierre Routy, Heiko Jessen, Anthony D Kelleher, Frederick Hecht, Rafick-Pierre Sekaly, Mary Carrington, Bruce D Walker, Todd M Allen, Eric S Rosenberg, Marcus Altfeld

**Affiliations:** 1 Ragon Institute of MGH, MIT, and Harvard, Massachusetts General Hospital, Boston, MA, USA; 2 Division of Infectious Diseases, Massachusetts General Hospital, Boston, MA, USA; 3 U.S. Military HIV Research Program, Walter Reed Army Institute of Research, Silver Spring, MD, USA; 4 Henry M. Jackson Foundation for the Advancement of Military Medicine, Bethesda, MD, USA; 5 Infectious Diseases Unit, University Medical Center Hamburg-Eppendorf, Hamburg, Germany; 6 Division of Hematology and Chronic Viral Illness Service MUHC, McGill University, Montreal, Quebec, Canada; 7 HIV Clinic Praxis Jessen, Berlin, Germany; 8 National Centre in HIV Epidemiology and Clinical Research, University of New South Wales, Darlinghurst, Australia; 9 Department of Medicine, San Francisco General Hospital, University of California, San Francisco, San Francisco, CA, USA; 10 Department of Microbiology and Immunology, Miller School of Medicine, University of Miami, Miami, FL, USA; 11 Cancer and Inflammation Program, Laboratory of Experimental Immunology, Leidos Biomedical Research, Inc., Frederick National Laboratories for Cancer Research, Frederick, MD, USA; 12 Howard Hughes Medical Institute, Chevy Chase, MD, USA; 13 Heinrich-Pette-Institut, Hamburg, Germany

**Keywords:** HLA-B*57, HLA-B, Acute HIV-1 infection, Primary HIV-1 infection, Viral load set point, MHC class I

## Abstract

**Background:**

HLA-B alleles are associated with viral control in chronic HIV-1 infection, however, their role in primary HIV-1 disease is unclear. This study sought to determine the role of HLA-B alleles in viral control during the acute phase of HIV-1 infection and establishment of the early viral load set point (VLSP).

**Findings:**

Individuals identified during primary HIV-1 infection were HLA class I typed and followed longitudinally. Associations between HLA-B alleles and HIV-1 viral replication during acute infection and VLSP were analyzed in untreated subjects. The results showed that neither HLA-B*57 nor HLA-B*27 were significantly associated with viral control during acute HIV-1 infection (Fiebig stage I-IV, n=171). HLA-B*57 was however significantly associated with a subsequent lower VLSP (p<0.001, n=135) with nearly 1 log_10_ less median viral load. Analysis of a known polymorphism at position 97 of HLA-B showed significant associations with both lower initial viral load (p<0.01) and lower VLSP (p<0.05). However, this association was dependent on different amino acids at this position for each endpoint.

**Conclusions:**

The effect of HLA-B*57 on viral control is more pronounced during the later stages of primary HIV-1 infection, which suggests the underlying mechanism of control occurs at a critical period in the first several months after HIV-1 acquisition. The risk profile of polymorphisms at position 97 of HLA-B are more broadly associated with HIV-1 viral load during primary infection and may serve as a focal point in further studies of HLA-B function.

## Findings

### Background

The course of HIV-1 infection exhibits remarkable variability in humans. While the mechanisms of natural HIV-1 control are still an area of active research, genetic studies have demonstrated the importance of human leukocyte antigen (HLA) alleles [1]. These immunogenetic effects have been discovered through large association studies, with the HLA-B alleles B*08, B*35, B*53, B*55, and B*56 associated with worse outcomes, while B*27 and B*57 are enriched in HIV controllers compared with non-controllers [2,3]. The majority of these studies have examined long-term outcomes in chronic HIV-1 infection. Currently, the role of HLA-B alleles in primary HIV-1 infection is not well described.

The HLA-B locus has extensive diversity in humans and it has been suggested that some alleles allow presentation of unique HIV-1-derived peptide epitopes to T lymphocytes which results in successful viral control [4]. This is evidenced by studies on T cell responses to the *gag*-derived KRWIILGLNK (KK10) peptide which is associated with the survival benefit of the B*27 allele [5]. Further insight into HIV-1 viral control was demonstrated by a study of genome-wide associations in “HIV-1 controllers”, individuals who maintained a viral load <2000 copies/mL for at least one year without anti-retroviral treatment (ART) [6]. Single nucleotide polymorphisms in position 97 of the peptide binding groove of HLA-B were found to be the most highly associated with HIV-1 controller status.

Our study poses the broad hypothesis that HLA-B alleles also play a role in the course of primary HIV-1 infection and establishment of the VLSP - focusing on the genetic factors known to have the greatest effect on viral control, i.e. HLA-B*57, HLA-B*27, and HLA-B position 97. We report that the protective effect of HLA-B*57 is more pronounced later in primary HIV-1 infection rather than during the acute phase. In contrast, polymorphisms at position 97 of HLA-B alleles were more broadly associated with HIV-1 viral control during primary infection.

## Methods

### Study subjects

A retrospective analysis of 428 individuals with primary HIV-1 infection was conducted from cohorts in North America, Germany and Australia (Massachusetts General Hospital, Boston, United States; Fenway Community Health Center, Boston, United States; AIDS Research Institute, University of California, San Francisco, United States; McGill University, Montreal, Canada; Infection network of the National Centre in HIV Epidemiology and Clinical Research, Darlinghurst, Australia; Jessen-Praxis, Berlin, Germany; and the University of California, San Diego, United States).

### Viral load testing

171 (40%) of the subjects were identified during acute HIV-1 infection Fiebig I-IV (defined by negative HIV-1 p24 antibody testing by ELISA with detectable HIV-1 RNA; or positive HIV-1 p24 antibody testing by ELISA and evolving (≤3 bands positive) HIV-1 Western Blot). The first viral load measurement for subjects with acute infection was used as the initial viral load and represented the peak viral load for that individual from the data available. VLSP could be calculated in 135 of the 171 acute subjects with longitudinal viral load testing (>4 measurements) for >6 months of untreated infection. VLSP was defined using the previously described algorithm by Fellay et al. [4]. The study was approved by the respective institutional review boards and informed consent was obtained for all subjects. This study was conducted in accordance with human experimentation guidelines of the Massachusetts General Hospital.

### HLA typing

High- and intermediate-resolution HLA class I typing was performed by sequence-specific PCR according to procedures described elsewhere [7].

### Statistical analysis

Wilcoxon rank-sum test was used for the comparison of two groups. The Kruskal–Wallis test with Dunn’s correction for multiple comparisons was used for comparing more than two groups. All P values are two-sided and P values of <0.05 were considered significant. Statistical analyses were conducted using GraphPad Prism (GraphPad Prism Software, La Jolla, CA).

## Results

Subjects with primary HIV-1 infection were recruited at seven clinical sites across three continents. Drawing on clinical history, HIV-1 Western blot/p24 testing, and longitudinal viral load data, we characterized primary HIV-1 infection in this large cohort. Among the 428 subjects with primary HIV-1 infection enrolled in the study, we identified 171 subjects diagnosed during acute HIV-1 infection (Fiebig I-IV). The median initial viral load in the 171 patients with acute infection was 450,000 copies/mL (IQR 170,000 - 750,001). Demographic analysis of this cohort is presented in Additional file 1: Table S1.

VLSP was calculated in 135 of these 171 individuals based on at least 4 measurements using an algorithm described by Fellay et al. [4]. VLSP could not be analyzed in the remaining 36 study subjects with acute infection due to anti-retroviral treatment (n=24), loss to follow-up (n=7), or if subjects had ≥2 viral load measurements which were ±2 SD of the mean and thus did not have a stable viral set point (n=5). Of the 135 subjects for whom VLSP was known, the median value was 22,082 copies/mL (IQR 5,883 - 53,558). These results confirm that the viral loads observed in our cohort are comparable with the course and kinetics of primary HIV-1 infection described in other studies [8].

**Figure 1:**
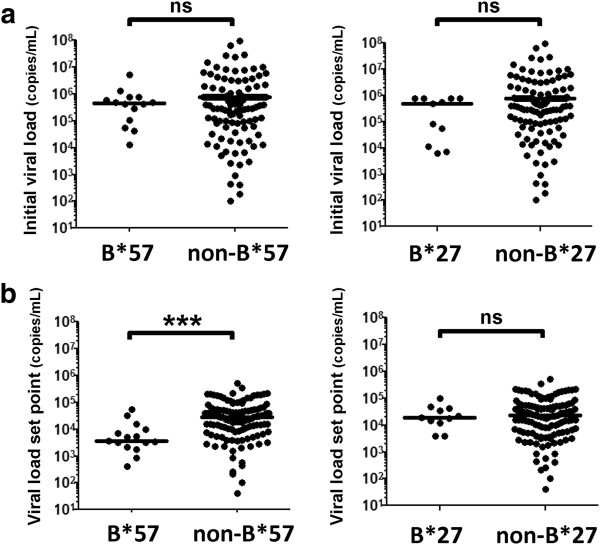
Effect of HLA-B*57 and HLA-B*27 on primary HIV-1 infection. (a) Initial viral load in subjects with acute HIV-1 infection (N=171) was analyzed based on the presence or absence of an HLA-B*57 or HLA-B*27 allele. **(b)** Viral load set point was determined in the subjects who remained in the study untreated for >6 months (N=135) and analyzed similarly. Statistical testing was done using Wilcoxon rank-sum (***p<0.001, ns non-significant).

We then investigated the protective effect of known HLA-B alleles during the course of primary HIV-1 infection. Individuals with HLA-B*57 and HLA-B*27 had similar initial viral loads as those with other alleles during acute infection (Figure 1a). However, HLA-B*57 was significantly associated with a lower VLSP (p<0.001) (Figure 1b). Prevalence of HLA-B*57 and HLA-B*27 was slightly higher in the VLSP group, however, this difference was not significant: HLA-B* 57: acute=14/171, VLSP=14/135 (p=0.55 Fisher's exact test); HLA-B*27: acute=11/171, VLSP=11/135 (p=0.67 Fisher's exact test). Detailed information about the genetic characterization of the cohort is presented in Additional file 2: Table S2. Thus, the major effect of HLA-B*57 on viral replication occurs later in primary HIV-1 infection at the time of the early VLSP, while the protective effect for HLA-B27 has been suggested to occur even later [9].

**Figure 2:**
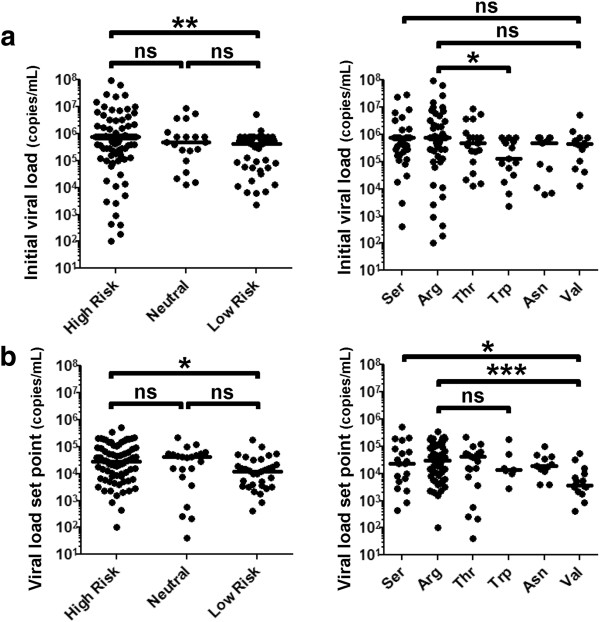
**Effect of the polymorphism at position 97 on primary HIV-1 infection.** Subjects were classified based on the amino acid at position 97 and assigned to a high, neutral, or low risk group based on OR shown in Table 1. Associations with **(a)** initial viral load during acute HIV-1 infection (N=171) and **(b)** VLSP (N=135) were determined. Statistical testing was done using Kruskal-Wallis with Dunn’s correction for multiple comparisons (*p<0.05, **p<0.01, ***p<0.001, ns non-significant).

Due to the delayed effect of HLA-B*57 and the implication for adaptive immune responses, we looked more broadly at risk factors for viral control within HLA-B. The polymorphism at position 97 of HLA-B has been shown to be one of the strongest predictors of viral control [6]. Therefore, we analyzed the imputed amino acids at position 97 in our primary HIV-1 cohort using HLA typing data and examined associations with viral load. Subjects were classified based on the amino acid at position 97 of their lowest risk allele, and then assigned into high, neutral, or low risk groups as shown in Table 1. Our results showed that subjects with a low risk polymorphism had significantly lower viral loads during acute HIV-1 infection, and this difference was associated with the tryptophan (Trp) at position 97 (Figure 2a). The low risk polymorphism group also had a significantly lower VLSP, which was associated with Val at position 97 (Figure 2b).

**Table 1:** Polymorphisms at position 97 of HLA-B and associated alleles

**Position 97**	**Risk profile**	OR viral control*	**Associated HLA-B Alleles**^**+**^
**S (Ser)**	**High risk**	0.5	B*07, B*08, B*40, B*48,
**R (Arg)**	**High risk**	0.6	B*35, B*15, B*53, B*51, B*40, B*44
**T (Thr)**	**Neutral**	1.2	B*55, B*13, B*51, B*52, B*73, B*56
**W (Trp)**	**Low risk**	1.9	B*14, B*5802, B*5806, B*5711
**N (Asn)**	**Low risk**	2.8	B*27, B*3702, B*1802, B*9529, B*4704
**V (Val)**	**Low risk**	5.6	B*57, B*5814, B*5514, B*4034, B*4030

*Odds ratio of HIV-1 Controller status [6].

^+^Bold indicates HLA-B alleles that were present in our cohort.

All individuals with Val at position 97 had the HLA-B*57 allele, therefore, we conducted a sensitivity analysis excluding individuals with HLA-B*57. Without HLA-B*57, the median VLSP between the groups with different amino acids at position 97 was not significantly different (p=0.84) but remained significant during acute infection (p=0.03). Taken together, our data demonstrate that amino acid polymorphisms at position 97 of HLA-B are broadly associated with viral control during primary HIV-1 infection, while HLA-B*57 has a later effect which is more pronounced at the time of the early VLSP.

## Discussion

The role of host genetics in HIV-1 infection continues to inform our understanding of HIV-1 pathogenesis. HLA-B*57 was first described as associated with a long-term non-progressive phenotype in chronic HIV-1 infection in 1996 [10], yet a clear understanding of how and why this allele contributes to the host response against viral replication still remains largely elusive. Previous studies including our own have implicated a role for HLA-B*57-restricted CD8^+^ T-cell responses in controlling viral replication [11,12].

Individuals with HLA-B*57 have been shown to have milder or absent symptoms during acute HIV-1 infection [11], which led us to question if this molecule might play a role through early innate mechanisms. In addition to the T-cell receptor (TCR), the HLA proteins interact directly with killer immunoglobulin-like receptors (KIRs) on natural killer cells which may represent another mechanism of HIV-1 control [13]. On the contrary, our data suggests that the major effect of HLA-B*57 occurs several weeks to months after infection, arguing for a role of adaptive immunity perhaps mediated by T-cell responses.

A limitation of our analysis is the lack of multiple viral load measurements during the acute phase, which is difficult to obtain in practice. However, the subjects in this study were entered at any point within the acute phase of the infection (Fiebig I-IV) and the enrollment was blinded to their HLA genotype. In addition, the Fiebig stage of each group did not differ significantly (Additional file 1: Table S1). Therefore, we believe the timing of the viral load measurements during acute infection are comparable between the groups, but this needs to be confirmed in other cohorts where the exact time of infection is known or can be more closely estimated.

It is important to note that our N of 171 individuals with acute HIV-1 infection gives 94% power to detect a 1 log_10_ difference in viral load between the HLA-B*57 and non-HLA-B*57 groups (2-sided Wilcoxon rank sum test with alpha=0.05), thus our threshold for significant differences between the groups was relatively high. It will be crucial to confirm these findings in larger cohorts with more viral load measurements to determine if HLA-B*57 may have a weaker than 1 log_10_ effect during acute HIV-1 infection. In addition, other well-described immunogenetic factors such as HLA-C haplotypes, homozygous/heterozygous genotypes, CCR5 variants, and KIR-HLA pairing are likely to be playing some role, however, could not be adequately assessed in our study given the sample size.

The polymorphism at position 97 of HLA-B alleles demonstrates how small changes MHC class I interactions can greatly influence the outcome of the immune response to HIV-1. Given the location of this amino acid deep in the binding pocket and comparing it with known structural data, it seems probable that the nature of this residue may impact peptide-TCR binding and the repertoire of presented viral epitopes [14]. Additional research on the biochemical and steric effects of the amino acid side chains at position 97 of HLA-B will be helpful in understanding how HLA class I molecules function in the natural immune response to HIV-1.

## Competing interests

The authors declare that they have no competing interests.

## Authors’ contributions

SAV, HS, and MA designed and performed experiments, analyzed and interpreted the data, and wrote the manuscript. NB, MG, and FP assisted with data interpretation and statistical analysis. DSK and MMA conducted analysis of viral load set point data. JR enrolled the study subjects and managed the clinical database. J-PR, HJ, ADK, FH, R-PS, enrolled study subjects and provided patient samples. MC assisted with HLA typing. TMA, BDW, and ESR enrolled study subjects and assisted with interpretation of data. All authors read and approved the final manuscript.

## Supplementary Material

Additional file 1: Table S1 Patient Characteristics of Primary HIV-1 Cohort. Click here for file

Additional file 2: Table S2 Genetic Categorization of Primary HIV-1 Cohort. Click here for file
