# Supplementary material for: Temporal effect of HLA-B*57 on viral control during primary HIV-1 infection
Source: Retrovirology. 2013 Nov 18;10:139. doi: 10.1186/1742-4690-10-139 (PMC3874665; doi:10.1186/1742-4690-10-139)
Supplement: Additional file 1: Table S1 — Patient Characteristics of Primary HIV-1 Cohort. [file 1742-4690-10-139-S1.doc]

| **Supplementary Table 1.**  **Patient Characteristics of Primary HIV-1 Cohort** | | | |
| --- | --- | --- | --- |
|  | |  |  |
| Number of subjects (N) | 171 | |  |
|  | |  |  |
| **Study Site** | |  |  |
| Boston, USA (N, %) | | 67 | 39% |
| Sydney, Australia (N, %) | | 43 | 25% |
| Berlin, Germany (N, %) | | 27 | 16% |
| Montreal, Canada (N, %) | | 23 | 13% |
| San Francisco, USA (N, %) | | 8 | 5% |
| San Diego, USA (N, %) | | 3 | 2% |
|  | |  |  |
| **Demographic** | |  |  |
| Age (years, median, IQR)* | | 36 | (31-52) |
| Sex (M/F,% male) | | 167/4 | 98% |
| Caucasian (%) | | 143 | 84% |
| Hispanic (%) | | 13 | 8% |
| Black (%) | | 7 | 4% |
| Asian (%) | | 5 | 3% |
| Other (%) | | 3 | 2% |
|  | |  |  |
| **Fiebig Staging** | |  |  |
| Entire cohort (median, IQR) | | 3 | (2-4) |
| B*57 (median, IQR) | | 3 | (3-4) |
| non-B*57 (median, IQR) | | 3 | (2-4) |
| B*27 (median, IQR) | | 3 | (3-4) |
| non-B*27 (median, IQR) | | 3 | (2-4) |

*Age data was not available for all study sites
