# Supplementary material for: Temporal effect of HLA-B*57 on viral control during primary HIV-1 infection
Source: Retrovirology. 2013 Nov 18;10:139. doi: 10.1186/1742-4690-10-139 (PMC3874665; doi:10.1186/1742-4690-10-139)
Supplement: Additional file 2: Table S2 — Genetic Categorization of Primary HIV-1 Cohort. [file 1742-4690-10-139-S2.doc]

| **Supplementary Table 2.**  **Genetic Categorization of Primary HIV-1 Cohort** | | | | | |
| --- | --- | --- | --- | --- | --- |
|  |  |  |  |  |  |
|  | **Acute (N=171)** | |  | **VLSP (N=135)** | |
|  |  |  |  |  |  |
|  | N | % of total |  | N | % of total |
|  |  |  |  |  |  |
| **HLA-B*57** | 14 | 8% |  | 14 | 10% |
| non HLA-B*57 | 157 | 92% |  | 121 | 90% |
|  |  |  |  |  |  |
| **HLA-B*27** | 11 | 6% |  | 11 | 8% |
| non HLA-B*27 | 160 | 94% |  | 124 | 92% |
|  |  |  |  |  |  |
| **Position 97 Risk** |  |  |  |  |  |
| High Risk | 111 | 65% |  | 81 | 60% |
| Neutral | 20 | 12% |  | 22 | 16% |
| Low Risk | 40 | 23% |  | 32 | 24% |
|  |  |  |  |  |  |
| **Position 97 AA** |  |  |  |  |  |
| Serine | 44 | 26% |  | 19 | 14% |
| Arginine | 67 | 39% |  | 62 | 46% |
| Threonine | 20 | 12% |  | 22 | 16% |
| Tryptophan | 15 | 9% |  | 7 | 5% |
| Asparagine | 11 | 6% |  | 11 | 8% |
| Valine | 14 | 8% |  | 14 | 10% |
